# Challenges of prescribing testosterone for sexual dysfunction in women: Number 7 – 2024

**DOI:** 10.61622/rbgo/2024FPS07

**Published:** 2024-07-26

**Authors:** Lucia Alves da Silva Lara, Joice Martins de Lima Pereira, Stany Rodrigues Campos de Paula, Flavia Fairbanks Lima de Oliveira, André Marquez Cunha, Théo Lerner, Yara Villar, Gabriela Pravatta Rezende Antoniassi, Cristina Laguna Benetti-Pinto

**Affiliations:** Departamento de Ginecologia e Obstetrícia Faculdade de Medicina Universidade de São Paulo Ribeirão Preto SP Brazil Departamento de Ginecologia e Obstetrícia, Faculdade de Medicina, Universidade de São Paulo, Ribeirão Preto, SP, Brazil.; Universidade Federal de Goiás Goiânia GO Brazil Universidade Federal de Goiás, Goiânia, GO, Brazil.; Departamento de Ginecologia e Obstetrícia Faculdade de Medicina Universidade de São Paulo Ribeirão Preto SP Brazil Departamento de Ginecologia e Obstetrícia, Faculdade de Medicina, Universidade de São Paulo, Ribeirão Preto, SP, Brazil.; Universidade Estadual de Campinas Campinas SP Brazil Universidade Estadual de Campinas, Campinas, SP, Brazil.; Universidade Federal de Goiás Goiânia GO Brazil Universidade Federal de Goiás, Goiânia, GO, Brazil.; Departamento de Ginecologia e Obstetrícia Faculdade de Medicina Universidade de São Paulo Ribeirão Preto SP Brazil Departamento de Ginecologia e Obstetrícia, Faculdade de Medicina, Universidade de São Paulo, Ribeirão Preto, SP, Brazil.; Departamento de Ginecologia e Obstetrícia Faculdade de Medicina Universidade de São Paulo Ribeirão Preto SP Brazil Departamento de Ginecologia e Obstetrícia, Faculdade de Medicina, Universidade de São Paulo, Ribeirão Preto, SP, Brazil.; Universidade Estadual de Campinas Campinas SP Brazil Universidade Estadual de Campinas, Campinas, SP, Brazil.; Universidade Estadual de Campinas Campinas SP Brazil Universidade Estadual de Campinas, Campinas, SP, Brazil.

## Key points

- Hypoactive sexual desire (HSD) is a condition in which a person presents absence or marked reduction of desire or motivation to engage in sexual activity.
- Testosterone (T) plays an important role in promoting sexual desire in women with reduced sexual desire after the onset of menopause.
- The mechanisms by which testosterone improves HSD are not yet well understood.
- There is no cutoff point for serum testosterone levels that would indicate androgen deficiency in women of any age.
- To date, there are no recommended serum testosterone levels to indicate or contraindicate its use in HSD.
- Off-label use of testosterone through various routes has been adopted and the evidence on this subject must be discussed.
- Testosterone absorption varies according to the skin type and the vehicle used in its formulation.

## Recommendations

- Measuring testosterone or other androgens is not indicated for the diagnosis of androgen deficiency.
- Consider prescribing testosterone in physiological doses for women after natural or surgical menopause or those with premature ovarian failure who present HSD without any other cause (dyadic, iatrogenic, psychic, etc.) and without contraindications to its use.
- During the reproductive period, including perimenopause, the prescription of testosterone in physiological doses is only indicated in the presence of HSD, and is not indicated for improving body composition or well-being.
- When prescribed, the transdermal route is preferred. The oral route should be avoided.
- As there are no industrialized products containing testosterone, manipulated testosterone is used. For this reason, serum testosterone measurement is recommended before starting use and after three months for the control and maintenance of physiological levels.
- If there is no improvement in sexual function within three to six months, its use should be discontinued.
- Available evidence points to low risk in using transdermal testosterone for up to three years.

## Background

Sexual function undergoes changes throughout a woman’s life. The prevalence of dysfunctions increases with advancing age, and is more marked after natural or surgical menopause, or due to oncological treatment. Relationships, psychosocial factors and comorbidities are important determinants of female sexuality, but the role of sexual steroids is increasingly being discussed, since ovarian failure can be associated with difficulty in arousal, orgasm and also with genitopelvic pain.^(1)^ The Women’s Health Across the Nation (SWAN) study,^(2,3)^ including more than 3,000 participants, annually evaluated domains related to women’s sexual function, such as the importance of sex and the frequency of sexual intercourse and masturbation, physical pleasure, emotional satisfaction, sexual desire, excitement and pain, with the aim to evaluate the impacts of menopausal transition on sexuality. A progressive increase in complaints of dyspareunia associated with vaginal dryness and reduced sexual desire was observed in the six prospective measurements, regardless of comorbidities, the woman’s biopsychosocial condition and menopausal symptoms, thereby valuing the association between sexual dysfunctions, aging and sex steroid deficit.^(2,3)^

Therefore, it is necessary to highlight the important role of steroids in female sexual function, whether as facilitators of sexual drive, in the trophic action on the genitalia, and in the synthesis and action of some neurotransmitters and neuropeptides involved in sexual response.^(4)^ Some characteristics of a woman’s expression of sexuality such as attractiveness, predisposition for sexual interaction^(5)^ and sexual motivation (desire)^(6)^ are also influenced by sexual hormones.

Among sexual steroids, estrogens are mainly responsible for female reproductive maturation, while androgens are considered metabolic, vascular and sexual hormones.^(7)^ The main biological forms of androgens in women are dehydroepiandrosterone sulfate (DHEAS), dehydroepiandrosterone (DHEA), androstenedione, testosterone (T) and dihydrotestosterone (DHT). In circulation, active testosterone is bound to albumin or in its free form; and it becomes inactive when bound to the sex hormone-binding globulin (SHBG).^(8)^

Several studies were carried out seeking to understand which steroid would be the most associated with female sexual dysfunction and how situations interfering with its levels could be corrected, finding evidence of a possible positive correlation between sexual desire and levels of free testosterone (r = 0.134), androstenedione (r = 0.199) and DHEA (r = 0.155) in women aged between 19 and 64 years.^(9)^ Previous studies have shown a risk for HSD and arousal dysfunction in women with DHEA levels below of the 10^th^ percentile,^(10)^ in addition to a reduction in thoughts and sexual frequency and a decrease in masturbation when there is a reduction in testosterone, free testosterone and DHT levels.^(11,12)^

Some authors have attempted to define an androgen deficiency syndrome in women, which would consist of symptoms such as fatigue, decreased well-being and low sexual interest. However, after a task force of exhaustive review of the criteria describing this syndrome, international entities took a stand against such a diagnosis due to the lack of standardization of the clinical picture and of serum testosterone values by age group, highlighting that circulating levels of androgens do not reflect the action on target tissues neither consider the individual sensitivity of peripheral receptors.^(13,14)^ Therefore, even though prescribing testosterone for women with changes in sexual desire is considered a therapeutic option, there is no cutoff point for serum testosterone levels in the female biological sex that define and characterize HSD or androgen deficiency. Furthermore, there are few countries in which the pharmaceutical industry provides testosterone formulations adjusted for women, with the justification that safety data for prescribing this hormone are insufficient. However, the informal testosterone market is a reality throughout the world, whether through the manipulation of formulas without standardization or through clandestine sales. The aim of this publication is to discuss some aspects of hormone therapy with prescription of testosterone in the treatment of HSD in women.

## What is the effect of testosterone on female sexual function?

Testosterone plays an important role in promoting female sexual desire^(15)^ through mechanisms not yet well understood. A systematic review and meta-analysis showed that testosterone improves sexual desire, arousal, the sensation of pleasure, the frequency of orgasm and the ability to respond to sexual stimulation, in addition to reducing suffering (distress) due to complaints related to sexual dysfunction in postmenopausal women with sexual desire dysfunction.^(16)^

In women, the ovaries, adrenal glands and peripheral tissues constitute the sources of androgens. Testosterone, directly or through metabolization into 5-alpha-dihydrotestosterone (DHT), by the action of the 5-alpha reductase enzyme or also by aromatization into estradiol, modulates biochemical and physiological mechanisms. Testosterone and DHT are the most potent androgens. Proportionally, serum testosterone derives mainly from ovarian secretion, while part is secreted by the adrenal gland,^(17)^ with cholesterol as its substrate. Dihydrotestosterone is produced from testosterone, metabolized by the action of the 5-alpha reductase enzyme.

It is known that the activity of testosterone occurs through the activation of its androgen receptors in the central nervous system and the genitalia, where they can be identified in the clitoris, labia minora and majora, vestibule and the vagina, among others, and in all layers of the mucosa, including the smooth muscle layer.^(18,19)^ In addition to the direct action on the androgen receptor, the effect of testosterone can be amplified by its conversion into DHT.^(20)^ Another activity of testosterone in the female body is its aromatization to estrogen.

Testosterone and its DHT metabolite modulate cellular function by genomic and nongenomic mechanisms. Through the genomic mechanism, testosterone or DHT bind to the androgen receptor by forming a complex that is activated and taken to the cell nucleus to exert its effect on target genes, promoting their modification to exert its action.^(21)^ The nongenomic action occurs through testosterone or DHT directly binding to the androgen receptor on the cell membrane of the target tissue.^(22)^ Furthermore, the action of testosterone can be prolonged by estrogen with evidence that the increase in estradiol increases the time that DHT acts in the medial preoptic area of the central nervous system, responsible for sexual behavior.^(7,23)^

Studies have demonstrated a trophic action of testosterone in the vagina, especially in the integrity and contractility of the non-vascular smooth muscle of the vaginal wall, its involvement in the neurovascular mechanism that promotes relaxation of the vascular smooth muscle, as well as in the preservation of the density of nerve fibers involved in neurotransmission of the stimuli that regulate the phases of excitation and lubrication.^(24)^ In animal models, testosterone promotes mucification of the vagina^(25)^ and favors lubrication by promoting an increase in local blood flow,^(26)^ which will facilitate the formation of transudate derived from the increase in blood pressure in the vaginal wall.^(27)^ In fact, a study of women undergoing breast cancer treatment using an aromatase inhibitor showed that the use of local testosterone for a period of four weeks resulted in an improvement in vaginal dryness, dyspareunia, pH and the maturation index of the vaginal epithelium,^(28)^ suggesting a direct action of testosterone in the vagina.

The physiology of female sexual dysfunction involves biopsychosocial interfaces, and the definitive etiology of HSD has not yet been fully clarified. Several models of sexual response are proposed, and the dual control model suggests that sexual response is mediated by stimulus and inhibition factors. From this perspective, the satisfactory sexual response is the result of the interaction of hormones and excitatory neurotransmitters with emphasis on testosterone, estradiol, dopamine and norepinephrine. Therefore, hypoactive desire could be a consequence of a deficiency in the production of these substances or an excess of inhibitory factors.^(29)^

## How to characterize HSD and what is its prevalence?

According to the criteria of the International Classification of Diseases (ICD-11), HSD dysfunction refers to a condition in which a person presents absence or a marked reduction in the desire or motivation to engage in sexual activity, manifested by any of the following criteria: i) reduced or absent desire (sexual thoughts or fantasies); ii) reduced or absent desire to respond to erotic signals and stimulation; or iii) inability to sustain the desire or interest in sexual activity once initiated, which are persistent, last several months and associated with clinically significant distress.^(30)^

The prevalence of HSD varies cross-culturally. A multicenter study involving European countries and the United States of America showed a prevalence of 11% of HSD in European women between 20 and 29 years of age and 53% among women aged between 60 and 70 years. In American women, the prevalence of HSD was 22% in those aged 20 to 29 years and 32% in those aged between 60 and 70 years.^(31)^ The PRESIDE study demonstrated that sexual complaints associated with suffering in American women ranged from 8.9% in the 18-44 years age group, 12.3% between 45 and 64 years, and 7.4% in women aged 65 and over.^(32)^

In a population survey in Brazil, the prevalence of reduced sexual desire in women ranged from 6.9% to 32.3%, showing a progressive increase with advancing age. In the age group of 18-39 years, the prevalence is lower, but reaches 9.0% between 40 and 49 years old, 14.9% between 50 and 59 years old, 25.7% between 60 and 69 years old and 32.3% in women aged 70 or over.^(33)^ However, the distress criterion was not assessed in this study, which still leaves doubt about the real prevalence of HSD in the Brazilian population.

## Is there an indication for prescribing testosterone for the treatment of female HSD?

Several randomized and controlled clinical studies show that testosterone with or without the addition of estrogen increases sexual desire and well-being in pre-, peri- and post-menopausal women, and in women with surgical menopause,^(34)^ although they have variable methodology and doses. Transdermal testosterone at a dose of 300 µg/day was associated with improvement in HSD, but this effect was not evident with the use of doses of 150 and 450 µg/day.^(34)^ A randomized and controlled study using the transdermal route in postmenopausal women aged 40-70 years also showed greater efficacy at a dose of 300 µg/day of testosterone in patches. After 24 weeks of use, there was a significant increase in the frequency of satisfactory sexual episodes in the treated group, compared to placebo.^(35)^ These data were replicated in hysterectomized women, demonstrating that this same dose (300 µg/day) of testosterone resulted in a 4.4-fold increase in testosterone and a five-fold increase in free testosterone in relation to pre-treatment levels.^(36)^

The positive results of the use of testosterone on sexual function derive from studies that used patches, although this technology is not available in many countries, such as Brazil. Therefore, it is worth mentioning other formulations tested in studies with smaller samples. Testosterone gel at a dose of 10 mg (in 1 g of hydroalcoholic gel) in association with estrogen therapy was used in 53 postmenopausal women and resulted in an increase in spontaneous sexual thoughts, fantasies, excitement and orgasm.^(37)^ This study was replicated in 70 postmenopausal women aged 40-60 years randomized to daily oral use of 1 mg of estradiol valerate in association with weekly use of 50 mg of testosterone in transdermal gel. There was a 25% increase in total testosterone and a significant improvement in sexual function measured by the female sexual function index (FSFI).^(38)^ Another study showed that the use of intravaginal testosterone propionate gel of silicone releasing 300 µg in women aged 40-70 years resulted in improved sexual desire, lubrication and dyspareunia.^(39)^ Sublingual testosterone on demand at a dose of 0.5 mg associated with 50 mg of sildenafil resulted in a significant increase in satisfactory sexual events and improved sexual response.^(40)^

Given the available evidence, testosterone has been indicated for the treatment of HSD in natural or surgical postmenopausal women undergoing oophorectomy, or with premature ovarian failure.^(4)^ Another publication, The International Society for the Study of Women’s Sexual Health Process of Care (ISSWSH), includes indications for perimenopausal women in their final reproductive years, and this recommendation is supported by the physiology of androgen decline and efficacy data, although based on weak evidence.^(4,7)^

Despite these data, to date Australia is the only country that offers a product containing testosterone for use in women. The American Food and Drug Administration (FDA) has not yet approved this product for women, considering that safety data are still insufficient. However, in the absence of testosterone-based products approved by government agencies, off-label testosterone has been widely used in women through products approved for men or formulations prescribed by doctors for manipulation, which are not standardized in terms of dose, vehicle used and frequency of use, and do not have product quality control.^(7)^ This implies an unpredictable risk to women’s health due to the lack of control over absorption and time of use. Injections, subcutaneous implants and cream or gel products that use similar doses to treat male hypogonadism are not recommended for use in women with perimenopausal and climacteric sexual dysfunctions given the significant risk of supraphysiological doses of testosterone, which can culminate in irreversible side effects and increased cardiovascular risk. Furthermore, manipulated formulas are not approved by the FDA or regulatory bodies in Brazil, which hinders the exact knowledge of the bioavailability, pharmacodynamics and pharmacokinetics of these medications.^(41)^

## How to prescribe testosterone for women with HSD?

Since there is a lack of product options suitable for women in conventional pharmacies, the only option currently available in Brazil for prescribing testosterone to women is through manipulation, and the transdermal route is preferred.^(42)^

The use of testosterone implants is not advised because it often results in supraphysiological testosterone levels as early as three months after implant insertion and long-term effects are unknown.^(43)^ Likewise, even though methyltestosterone in combination with conjugated equine estrogen via oral demonstrates improvement in sexual desire, it is not recommended both because it maintains supraphysiological blood levels of testosterone and causes liver toxicity.^(44,45)^

Therefore, considering the available evidence, for starting HSD treatment in women, the recommendation is a daily dose of 300 µg transdermal testosterone^(34)^ in patches to maintain effective physiological levels. The dose can be increased up to 5 mg per day, depending on clinical response, always using the concept of the lowest effective dose.^(46-48)^ However, patches are not available in Brazil, and the most practical way of prescribing for manipulation is in gel or cream.

Some variables can affect the quality of manipulated preparations when prescribing a formula. The percutaneous passage of testosterone is limited by the barrier of the organized stratum corneum, the molecular weight of testosterone and the characteristics of the vehicle used to carry it.^(49)^ Considering the vehicle to be used, Pentravan^®^ (P) is a water-soluble and fat-soluble vehicle with high permeation precision performance, which acts at pH levels from 2 to 12. It is hypoallergenic and can be applied to mucous membranes and skin. The phospholipid nanosome composition of P carries the drug through the skin, favoring the permeation of the molecule to the dermis, where it will be taken into the bloodstream. Its composition contains phospholipids that confer its characteristic odor and favor the performance and gradual permeation of testosterone, resulting in 55% use of the applied dose.^(50)^ In in vitro studies with the skin of men and women, P showed good performance in promoting the penetration and absorption of testosterone, achieving a penetration peak 4.2 times greater at 10 hours in relation to the first hour of skin exposure to 10% testosterone.^(51)^ When using the vulvovaginal route, testosterone at a dose of 3 mg/mL in P gel resulted in a significant increase in blood testosterone concentrations that went from a basal level of 20 ± 15 ng/dL to 312 ± 264 ng/dL three hours after application. After 12 hours, serum testosterone dropped rapidly to 67 ± 40 ng/dL, returning to baseline levels 24 hours after exposure.^(52)^

On the other hand, when using the alcoholic vehicle to prescribe testosterone, it is generally equipped with other components to ensure better drug absorption. According to the manufacturer’s leaflet for the compound containing testosterone 10 mg/g, ethanol accounts for 96% of the excipient, which also contains isopropyl myristate, ethanol, sodium hydroxide and purified water, allowing an absorption of 9% to 14% of testosterone, significantly lower compared to P.^(53)^ Although studies on the bioavailability of testosterone in an alcoholic vehicle in women are still limited, the testosterone available in Australia for use in women is prepared in an alcoholic vehicle.^(54)^

When considering all the variables affecting the final availability of testosterone, it is not possible to guarantee that these formulas will result in an adequate dose for the woman. Therefore, base testosterone in suggested doses of 1-5 mg/mL for the management of female HSD needs to be adjusted according to the vehicle used (Table 1). For example; in high-performance vehicles, base testosterone at a dose of 5 mg in 1 mL of P will result in a minimum absorption of 2,500 µ,^(50)^ which corresponds to a five times greater dose than that needed by a woman.^(50)^ Base testosterone at a dose of 5 mg in 1 mL of the alcoholic vehicle will result in a minimum absorption of 450 µ. Therefore, lower doses are suggested for high-performance vehicles and larger doses for alcoholic vehicles.^(55)^ It is also suggested that 1 dose/pump releases 1 mL of the formula for application on thighs, buttocks or lower abdomen, avoiding the mammary lymphatic system. It is recommended that the woman starts therapy and follow-up on a serial basis in order to track possible effects of excess androgens and evaluate the response to treatment. This prescription should be suspended if there is no improvement in symptoms within six months due to the possible association of another cause for sexual dysfunction.^(45,56)^ A baseline serum dosage of total testosterone and another after three to four weeks of daily use are recommended to adjust the dose. Once the dose is maintained, reevaluate after three months. For women in the reproductive period, in the presence of serum levels within the physiological range, total testosterone measurements can be carried out every six months,^(56)^ as long as the same vehicle and the indicated location for manipulation are maintained while continuing its use.

**Table 1:** Testosterone bioavailability for women according to the vehicle used

Dose	Vehicle	Absorption	Prescription example
Testosterone base 1-5 mg	High performance vehicle (Pentravan^®^)	50% to 63%	Testosterone base.....2.0 mg Pentravan^®^ q.s.......1.0 mL
	Alcoholic vehicle	9% to 14%	Testosterone base.....5.0 mg Alcoholic vehicle q.s....1.0 mL

## What are the main aspects related to testosterone absorption?

The influence of several aspects must be considered when indicating the exogenous use of testosterone. Testosterone absorption is influenced by factors such as route of administration, skin thickness, pH at the site of application, type of vehicle used for its dilution, site of administration, in addition to individual variation in aromatase levels that can amplify the conversion of testosterone into estrogens, but also into DHT, which can, especially at the site of application, increase hair growth.^(57)^

Oral, intramuscular, vaginal and transdermal administration routes have already been tested in postmenopausal women (natural and surgical). When comparing different administration sites, the absorption of topical testosterone in the vulva was greater in relation to application on the skin of the upper limb.^(58)^ This finding was not replicated in women of reproductive age, thereby suggesting that changes in the skin after menopause in association with changes due to advancing age may influence the absorption of testosterone. The thickness of the skin influences absorption, and thinner skin is more favorable to greater testosterone absorption.^(42)^ An interesting fact is that the presence of hair and the use of antiperspirant deodorants do not influence the absorption of testosterone applied to the axilla.^(59)^

Thus, transdermal, percutaneous, oral and intravaginal routes seem to increase the sexual function of perimenopausal and climacteric women, but the first two end up being the main choices given their greater safety. However, consider the particular importance of all these factors identified as modifiers of absorption in the use of products required for manipulation, which indicates periodic control of serum testosterone levels throughout the period of use.

## What are the precautions for applying testosterone to the skin?

For optimal absorption of the drug, the skin must be dry and kept dry for a period of two to six hours after application. When the skin is wet after applying testosterone gel, there is an 81% reduction in the amount of testosterone availability and a 10%-14% reduction in absorption.^(60)^ Care is needed regarding contact between children and adults, skin-to-skin contact or even with a barrier on the areas that received testosterone. Care in the contact of children and adults with areas that received testosterone is necessary because of the probability of drug absorption upon skin-to-skin contact or even with a tissue barrier.^(61)^ When skin exposure is ceased, testosterone returned to basal levels after 48 hours.

## What are the adverse effects of testosterone?

The most common effects in women using testosterone at physiological levels are acne, oily skin and hair growth.^(16)^ No serious adverse events have been demonstrated when testosterone therapy was used at doses reaching physiological levels found in women in the reproductive phase.^(16)^

High-dose testosterone and its esters are used in transgender men to promote the appearance of desired masculine characteristics and constitute an important model for evaluating the beneficial and adverse effects of testosterone use on the female body with intact and functioning ovaries. The literature provides data on up to 17 years of follow-up of trans men using testosterone undecanoate. Changes in the lipid profile were observed, with a reduction in HDL-c and an increase in LDL-c and triglycerides, without an increase in the risk of death in the younger population.^(62)^ However, long-term effects, when this population reaches the age of greatest cardiovascular risk, are unknown. Note that virilizing testosterone levels contribute to suppress circulating insulin concentrations and increase proteins in the insulin signaling pathway in the liver and the altered phosphorylation of key proteins in the control of insulin sensitivity, suppressing insulin signaling in adipose white tissue.^(63)^

## When is vulvar testosterone indicated?

The vulvar use of testosterone derives from the treatment of lichen and is based on the higher concentration of androgen receptors in the vulva. However, there is no randomized and controlled study to test this formulation for the treatment of HSD. The dose used in the formulations is 2% testosterone propionate in petrolatum and has been used in the vulva by applying to the labia minora, clitoris and vaginal introitus.^(64)^ Some studies have evaluated the use of vaginal testosterone to treat the Genitourinary Syndrome of Menopause (GSM) and dyspareunia, although with a low number of cases and without important effects in relation to improving sexual desire.^(65,66)^

There are studies evaluating the administration of 2 mg of testosterone propionate vaginally and showing different absorption. A peak in serum levels of total and free testosterone was observed after six hours of application by this route in a population of ten healthy women aged between 20 and 40 years.^(67)^ Testosterone propionate at a dose of 2 mg in neutral intravaginal cream promoted a maximum serum testosterone peak of 7.71 nmol/L 5.5 hours after exposure to testosterone, against 2.99 nmol/L during placebo use, although the peak testosterone did not result in increased genital and central sexual response.^(67)^

In contrast, although the topical use of estrogens is the gold standard for the treatment of GSM, studies with vaginal DHEA preparations for this purpose have been carried out with data demonstrating improvement in dyspareunia and vaginal atrophy, in addition to improved sexual function. There is a formulation available in some countries (not in Brazil) for purchase in pharmacies in the format of vaginal ovules containing DHEA. Safety data for its use in specific conditions, such as in women with breast cancer are still unavailable.^(65,68,69)^

## Conclusion

The use of transdermal testosterone in climacteric women may result in improved sexual function in women with HSD. Other causes of this dysfunction should be evaluated and considered before prescribing testosterone. Given the lack of formulations available on the market, women who use this therapy need to be advised regarding the lack of safety data on manipulated formulas in relation to bioavailability, pharmacokinetics and pharmacodynamics, in addition to the lack of long-term studies. It is recommended to measure baseline testosterone levels before starting treatment and after three to six weeks, in addition to maintaining a routine clinical and laboratory evaluation in a serial manner, monitoring possible effects of excess androgens. Regarding GSM, the use of vaginal DHEA seems to present good results in relation to dyspareunia and vaginal atrophy, although data to support universal recommendations are still lacking.

### National Commission Specialized in Sexology of the Brazilian Federation of Gynecology and Obstetrics Associations (Febrasgo)

President: Lucia Alves da Silva Lara

Vice-president: Sandra Cristina Poerner Scalco

Secretary: Flavia Fairbanks Lima de Oliveira

Members:

André Marquez Cunha

Andrea Cronemberger Rufino

Carmita Helena Abdo

Eduardo Siqueira Fernandes

Fabiene Bernardes Castro Vale

Gerson Pereira Lopes

Joice Martins de Lima Pereira

Siglia Sousa de França

Stany Rodrigues Campos de Paula

Suzane Beirao de Almeida

Sylvia Maria Oliveira da Cunha Cavalcanti

Theo Lerner

Yara Maia Villar de Carvalho

Rodrigo Itocazo Rocha

## References

[1] Clayton AH, Valladares Juarez EM (2017). Female sexual dysfunction. Psychiatr Clin North Am.

[2] Avis NE, Brockwell S, Randolph JF, Shen S, Cain VS, Ory M (2009). Longitudinal changes in sexual functioning as women transition through menopause: results from the Study of Women's Health Across the Nation. Menopause.

[3] Avis NE, Zhao X, Johannes CB, Ory M, Brockwell S, Greendale GA (2005). Correlates of sexual function among multi-ethnic middle-aged women: results from the Study of Women's Health Across the Nation (SWAN). Menopause.

[4] Goldstein I, Kim NN, Clayton AH, DeRogatis LR, Giraldi A, Parish SJ (2017). Hypoactive sexual desire disorder: International Society for the Study of Women's Sexual Health (ISSWSH) Expert Consensus Panel Review. Mayo Clin Proc.

[5] Puts DA, Bailey DH, Cárdenas RA, Burriss RP, Welling LL, Wheatley JR (2013). Women's attractiveness changes with estradiol and progesterone across the ovulatory cycle. Horm Behav.

[6] Sherwin BB, Gelfand MM, Brender W (1985). Androgen enhances sexual motivation in females: a prospective, crossover study of sex steroid administration in the surgical menopause. Psychosom Med.

[7] Parish SJ, Simon JA, Davis SR, Giraldi A, Goldstein I, Goldstein SW (2021). International Society for the Study of Women's Sexual Health Clinical Practice Guideline for the use of systemic testosterone for hypoactive sexual desire disorder in women. J Sex Med.

[8] Smith T, Batur P (2021). Prescribing testosterone and DHEA: the role of androgens in women. Cleve Clin J Med.

[9] Wåhlin-Jacobsen S, Pedersen AT, Kristensen E, Laessøe NC, Lundqvist M, Cohen AS (2015). Is there a correlation between androgens and sexual desire in women?. J Sex Med.

[10] Davis SR, Davison SL, Donath S, Bell RJ (2005). Circulating androgen levels and self-reported sexual function in women. JAMA.

[11] Riley A, Riley E (2000). Controlled studies on women presenting with sexual drive disorder: I. Endocrine status. J Sex Marital Ther.

[12] Van Goozen SH, Wiegant VM, Endert E, Helmond FA, Van de Poll NE (1997). Psychoendocrinological assessment of the menstrual cycle: the relationship between hormones, sexuality, and mood. Arch Sex Behav.

[13] Wierman ME, Arlt W, Basson R, Davis SR, Miller KK, Murad MH (2014). Androgen therapy in women: a reappraisal: an Endocrine Society clinical practice guideline. J Clin Endocrinol Metab.

[14] Simpson ER (2002). Aromatization of androgens in women: current concepts and findings. Fertil Steril.

[15] Nappi RE, Tiranini L, Martini E, Bosoni D, Righi A, Cucinella L (2022). Medical treatment of female sexual dysfunction. Urol Clin North Am.

[16] Islam RM, Bell RJ, Green S, Page MJ, Davis SR (2019). Safety and efficacy of testosterone for women: a systematic review and meta-analysis of randomised controlled trial data. Lancet Diabetes Endocrinol.

[17] Cussen L, McDonnell T, Bennett G, Thompson CJ, Sherlock M, O'Reilly MW (2022). Approach to androgen excess in women: clinical and biochemical insights. Clin Endocrinol (Oxf).

[18] Hodgins MB, Spike RC, Mackie RM, MacLean AB (1998). An immunohistochemical study of androgen, oestrogen and progesterone receptors in the vulva and vagina. Br J Obstet Gynaecol.

[19] Shapiro E, Huang HY, Wu XR (2000). Uroplakin and androgen receptor expression in the human fetal genital tract: insights into the development of the vagina. J Urol.

[20] Fassnacht M, Schlenz N, Schneider SB, Wudy SA, Allolio B, Arlt W (2003). Beyond adrenal and ovarian androgen generation: increased peripheral 5 alpha-reductase activity in women with polycystic ovary syndrome. J Clin Endocrinol Metab.

[21] Chang C, Lee SO, Wang RS, Yeh S, Chang TM (2013). Androgen receptor (AR) physiological roles in male and female reproductive systems: lessons learned from AR-knockout mice lacking AR in selective cells. Biol Reprod.

[22] Celec P, Ostatníková D, Hodosy J (2015). On the effects of testosterone on brain behavioral functions. Front Neurosci.

[23] Roselli CE, Resko JA (1993). Aromatase activity in the rat brain: hormonal regulation and sex differences. J Steroid Biochem Mol Biol.

[24] Maseroli E, Vignozzi L (2020). Testosterone and vaginal function. Sex Med Rev.

[25] Kennedy TG (1974). Vaginal mucification in the ovariectomized rat in response to 5alpha-pregnane-3,20-dione, testosterone and 5alpha-androstan-17beta-ol-3-one: test for progestogenic activity. J Endocrinol.

[26] Heard-Davison A, Heiman JR, Kuffel S (2007). Genital and subjective measurement of the time course effects of an acute dose of testosterone vs. placebo in postmenopausal women. J Sex Med.

[27] Paavonen J (1983). Physiology and ecology of the vagina. Scand J Infect Dis Suppl.

[28] Witherby S, Johnson J, Demers L, Mount S, Littenberg B, Maclean CD (2011). Topical testosterone for breast cancer patients with vaginal atrophy related to aromatase inhibitors: a Phase I/II Study. Oncologist.

[29] Pettigrew JA, Novick AM (2021). Hypoactive sexual desire disorder in women: physiology, assessment, diagnosis, and treatment. J Midwifery Womens Health.

[30] ICD-11 for Mortality and Morbidity Statistics (2022). https://icd.who.int/dev11/l-m/en#/http://id.who.int/icd/entity/1189253773.

[31] Hayes RD, Dennerstein L, Bennett CM, Koochaki PE, Leiblum SR, Graziottin A (2007). Relationship between hypoactive sexual desire disorder and aging. Fertil Steril.

[32] Parish SJ, Hahn SR (2016). Hypoactive sexual desire disorder: a review of epidemiology, biopsychology, diagnosis, and treatment. Sex Med Rev.

[33] Abdo CH, Valadares AL, Oliveira WM, Scanavino MT, Afif-Abdo J (2010). Hypoactive sexual desire disorder in a population-based study of Brazilian women: associated factors classified according to their importance. Menopause.

[34] Ganesan K, Habboush Y, Sultan S (2018). Transdermal testosterone in female hypoactive sexual desire disorder: a rapid qualitative systematic review using grading of recommendations assessment, development and evaluation. Cureus.

[35] Davis SR, Moreau M, Kroll R, Bouchard C, Panay N, Gass M (2008). Testosterone for low libido in postmenopausal women not taking estrogen. N Engl J Med.

[36] Simon J, Braunstein G, Nachtigall L, Utian W, Katz M, Miller S (2005). Testosterone patch increases sexual activity and desire in surgically menopausal women with hypoactive sexual desire disorder. J Clin Endocrinol Metab.

[37] Nathorst-Böös J, Flöter A, Jarkander-Rolff M, Carlström K, von Schoultz B (2006). Treatment with percutanous testosterone gel in postmenopausal women with decreased libido--effects on sexuality and psychological general well-being. Maturitas.

[38] Chaikittisilpa S, Soimongkol K, Jaisamrarn U (2019). Efficacy of oral estrogen plus testosterone gel to improve sexual function in postmenopausal women. Climacteric.

[39] Fernandes T, Costa-Paiva LH, Pinto-Neto AM (2014). Efficacy of vaginally applied estrogen, testosterone, or polyacrylic acid on sexual function in postmenopausal women: a randomized controlled trial. J Sex Med.

[40] Tuiten A, van Rooij K, Bloemers J, Eisenegger C, van Honk J, Kessels R (2018). Efficacy and safety of on-demand use of 2 treatments designed for different etiologies of female sexual interest/arousal disorder: 3 randomized clinical trials. J Sex Med.

[41] Sood R, Shuster L, Smith R, Vincent A, Jatoi A (2011). Counseling postmenopausal women about bioidentical hormones: ten discussion points for practicing physicians. J Am Board Fam Med.

[42] van de Sandt JJ, van Burgsteden JA, Cage S, Carmichael PL, Dick I, Kenyon S (2004). In vitro predictions of skin absorption of caffeine, testosterone, and benzoic acid: a multi-centre comparison study. Regul Toxicol Pharmacol.

[43] Davis SR, McCloud P, Strauss BJ, Burger H (1995). Testosterone enhances estradiol's effects on postmenopausal bone density and sexuality. Maturitas.

[44] Lobo RA, Rosen RC, Yang HM, Block B, Van Der Hoop RG (2003). Comparative effects of oral esterified estrogens with and without methyltestosterone on endocrine profiles and dimensions of sexual function in postmenopausal women with hypoactive sexual desire. Fertil Steril.

[45] Vegunta S, Kling JM, Kapoor E (2020). Androgen therapy in women. J Womens Health (Larchmt).

[46] Krapf JM, Simon JA (2017). A sex-specific dose-response curve for testosterone: could excessive testosterone limit sexual interaction in women?. Menopause.

[47] Weiss RV, Hohl A, Athayde A, Pardini D, Gomes L, Oliveira M (2019). Testosterone therapy for women with low sexual desire: a position statement from the Brazilian Society of Endocrinology and Metabolism. Arch Endocrinol Metab.

[48] Mason KA, Schoelwer MJ, Rogol AD (2020). Androgens during infancy, childhood, and adolescence: physiology and use in clinical practice. Endocr Rev.

[49] Bronaugh RL, Franz TJ (1986). Vehicle effects on percutaneous absorption: in vivo and in vitro comparisons with human skin. Br J Dermatol.

[50] Fagron (2022). Pentravan ®.

[51] Lehman PA, Raney SG (2012). In vitro percutaneous absorption of ketoprofen and testosterone: comparison of pluronic lecithin organogel vs. pentravan cream. Int J Pharm Compd.

[52] Maia HM, Haddad C, Maia R, França CE, Casoy J (2013). Pulsatile administration of testosterone by the vaginal route using Pentravan.

[53] Gooren LJ, Bunck MC (2003). Transdermal testosterone delivery: testosterone patch and gel. World J Urol.

[54] El-Hage G, Eden JA, Manga RZ (2007). A double-blind, randomized, placebo-controlled trial of the effect of testosterone cream on the sexual motivation of menopausal hysterectomized women with hypoactive sexual desire disorder. Climacteric.

[55] Lawley Pharmaceuticals Pty (2023). Androfeme ^®^ 1.

[56] Davis SR, Baber R, Panay N, Bitzer J, Perez SC, Islam RM (2019). Global consensus position statement on the use of testosterone therapy for women. J Clin Endocrinol Metab.

[57] Heylings JR, Davies DJ, Burton R (2018). Dermal absorption of testosterone in human and pig skin in vitro. Toxicol In Vitro.

[58] Oriba HA, Bucks DA, Maibach HI (1996). Percutaneous absorption of hydrocortisone and testosterone on the vulva and forearm: effect of the menopause and site. Br J Dermatol.

[59] Small DS, Ni X, Polzer P, Vart R, Satonin DK, Mitchell MI (2014). Effect of deodorant and antiperspirant use and presence or absence of axillary hair on absorption of testosterone 2% solution applied to men's axillae. J Sex Med.

[60] Stahlman J, Britto M, Fitzpatrick S, McWhirter C, Testino SA, Brennan JJ (2012). Effects of skin washing on systemic absorption of testosterone in hypogonadal males after administration of 1.62% testosterone gel. Curr Med Res Opin.

[61] Stahlman J, Britto M, Fitzpatrick S, McWhirter C, Testino SA, Brennan JJ (2012). Serum testosterone levels in non-dosed females after secondary exposure to 1.62% testosterone gel: effects of clothing barrier on testosterone absorption. Curr Med Res Opin.

[62] Velho I, Fighera TM, Ziegelmann PK, Spritzer PM (2017). Effects of testosterone therapy on BMI, blood pressure, and laboratory profile of transgender men: a systematic review. Andrology.

[63] Kothmann KH, Jacobsen V, Laffitte E, Bromfield C, Grizzaffi M, Jarboe M (2021). Virilizing doses of testosterone decrease circulating insulin levels and differentially regulate insulin signaling in liver and adipose tissue of females. Am J Physiol Endocrinol Metab.

[64] Ayhan A, Guven S, Guvendag Guven ES, Sakinci M, Gultekin M, Kucukali T (2007). Topical testosterone versus clobetasol for vulvar lichen sclerosus. Int J Gynaecol Obstet.

[65] Davis SR, Robinson PJ, Jane F, White S, White M, Bell RJ (2018). Intravaginal testosterone improves sexual satisfaction and vaginal symptoms associated with aromatase inhibitors. J Clin Endocrinol Metab.

[66] Nappi RE, Tiranini L, Martini E, Bosoni D, Righi A, Cucinella L (2022). Medical treatment of female sexual dysfunction. Urol Clin North Am.

[67] Apperloo M, Midden M, van der Stege J, Wouda J, Hoek A, Weijmar Schultz W (2006). Vaginal application of testosterone: a study on pharmacokinetics and the sexual response in healthy volunteers. J Sex Med.

[68] Sarmento AC, Costa AP, Lírio J, Eleutério J, Baptista PV, Gonçalves AK (2022). Efficacy of hormonal and nonhormonal approaches to vaginal atrophy and sexual dysfunctions in postmenopausal women: a systematic review. Rev Bras Ginecol Obstet.

[69] Aziz A, Brännström M, Bergquist C, Silfverstolpe G (2005). Perimenopausal androgen decline after oophorectomy does not influence sexuality or psychological well-being. Fertil Steril.

